# Land-use classification based on high-resolution remote sensing imagery and deep learning models

**DOI:** 10.1371/journal.pone.0300473

**Published:** 2024-04-18

**Authors:** Mengmeng Hao, Xiaohan Dong, Dong Jiang, Xianwen Yu, Fangyu Ding, Jun Zhuo

**Affiliations:** 1 Institute of Geographic Sciences and Natural Resources Research, Chinese Academy of Sciences, Beijing, China; 2 College of Resources and Environment, University of Chinese Academy of Sciences, Beijing, China; 3 ByteDance Inc, Beijing, China; Hainan Normal University, CHINA

## Abstract

High-resolution imagery and deep learning models have gained increasing importance in land-use mapping. In recent years, several new deep learning network modeling methods have surfaced. However, there has been a lack of a clear understanding of the performance of these models. In this study, we applied four well-established and robust deep learning models (FCN-8s, SegNet, U-Net, and Swin-UNet) to an open benchmark high-resolution remote sensing dataset to compare their performance in land-use mapping. The results indicate that FCN-8s, SegNet, U-Net, and Swin-UNet achieved overall accuracies of 80.73%, 89.86%, 91.90%, and 96.01%, respectively, on the test set. Furthermore, we assessed the generalization ability of these models using two measures: intersection of union and F1 score, which highlight Swin-UNet’s superior robustness compared to the other three models. In summary, our study provides a systematic analysis of the classification differences among these four deep learning models through experiments. It serves as a valuable reference for selecting models in future research, particularly in scenarios such as land-use mapping, urban functional area recognition, and natural resource management.

## 1. Introduction

Land-use mapping based on remote sensing imagery has become increasingly important in providing essential information for many applications, such as urban planning [1, 2], ecological management [3, 4], regional design of crop cultivation [5, 6] and environmental assessment [7]. With the rapid development of remote sensing technologies, the spatial resolution of imagery has sharply increased over the past decade, providing opportunities to extract more precise land-cover information [8, 9]. However, this development also renders information extraction of high spatial resolution (HSR) imagery more challenging due to the low interclass disparity and high interclass variability in HSR imagery [10].

Traditionally, land-cover mapping methods, such as maximum likelihood (ML), decision tree (DT), artificial neural network (ANN), support vector machine (SVM), or random forest (RF), are always operated at the pixel level. Using pixel-based classification on Thematic Mapper (TM) data, Huang et al. [11] compared SVM with three other popular machine learning classifiers (i.e., ML, NN and DT), revealing that SVM achieved a higher level of classification accuracy than the other three algorithms. Pal and Mather [12] adopted three machine learning algorithms to classify Landsat-7 Enhanced Thematic Mapper Plus (ETM+) data into seven land cover types (i.e., wheat, potato, peas or beans), illustrating that SVMs performed better than ML and ANNs during pixel-based classification. Based on Landsat-7 ETM+ imagery, Pal [13] used RF and SVM classification to identify seven different land covers at the pixel level, showing that the classification accuracy achieved by RFs is equal to that generated by SVMs. Based on Landsat TM and ETM+ data, Otukei et al. [14] compared three classification algorithms using pixel-based image analysis and found that DT performed better than both ML and SVMs. However, per-pixel classification approaches can easily produce a similarly speckled “salt-and-pepper” appearance [15].

Utilizing spectral, textural and shape information of image objects, object-based classifications can obtain more precise information on land cover than classic pixel-based classification methods [16–18]. Based on an image with 15m spatial resolution, pixel-based and object-based image classification approaches for land-cover mapping in a coal fire area were compared by Yan et al.; the results suggested that the thematic map derived from object-based image analysis obtained a much higher accuracy than that generated by the pixel-based approach [19]. Using SPOT-5 imagery, Duro et al. employed pixel-based and object-based image analysis with the same machine learning algorithms to identify agricultural landscapes and found that the latter offered a more contiguous depiction of land cover than the former [15]. Based on QuickBird image data, Myint et al. [20] extracted urban land cover using both per-pixel classifiers and object-based classifiers; the results revealed that the object-based image analysis approach performed significantly better than traditional per-pixel classifications. Note that the object-based classification prototype starts with partitioning imagery into spatially contiguous groups of pixels generally through a multiresolution segmentation algorithm. However, there is no widely accepted method available to determine the optimal scale for classification, requiring a process of trial and error experiments [20, 21].

In recent years, deep learning techniques, i.e., convolutional neural networks (CNNs), have achieved remarkable success in image interpretation, discovering intrinsic features with deeper hierarchical neural networks [22–28]. For example, CNN-based methods are usually employed to classify whether an HSR image patch contains target features, including vehicles, ships, bridges and airplanes [29–32]. In addition, several CNN-based scene classification frameworks were adopted to identify land-cover types using HSR image patches [10, 33–35]. These patch-based CNN classification procedures are conducted in an “image-label” manner, while remote sensing image classification expects a “pixel-label” model. To obtain a class map with the same dimensions as the original image, the fully convolutional network (FCN) model, which replaces fully connected layers with convolutional layers, was proposed by Long et al. [36]. Fu et al. [37] compared the FCN approach with both object-based classification and patch-based CNN classification on the same HRS images; the experiments illustrated that FCN-based classification yielded an obvious improvement in accuracy. Currently, there are several new bottleneck architectures (i.e., SegNet and U-Net), extracting land cover for HRS imagery through an end-to-end approach [38, 39]. For instance, the SegNet model was selected to delineate the fields of smallholder farms from satellite imagery [40], and the U-Net model was applied to cloud detection in remote sensing images [41]. At the same time, another transformer based deep learning models have also shown strong advantages in land use classification. For example, Yao et al. completed 10 class segmentation of Sentinel-2 MSI images at a resolution of 10 meters based on the Swin-UNet deep learning model [42].

With the improvement of spatial resolution of remote sensing image and the complexity of details, many deep learning classification methods have emerged and been applied in remote sensing image classification. This study compares the performance of four well-established and robust deep learning models (FCN-8s, SegNet, U-Net, and Swin-UNet) using open benchmark high-resolution remote sensing datasets. Compared with other research papers with comparative models, this study has the following advantages: (1) The spatial resolution of the dataset selected in this study have reached submeter level, and the details of ground objects are complex and diverse, which is in line with the development trend of current remote sensing images. (2) This study uses multiple evaluation indicators to evaluate four types of highly representative models from two perspectives: index evaluation and visual evaluation. Through this study, on the one hand, it can solve the current confusion about the differences between remote sensing image classification models, and on the other hand, it can provide reference for model selection in land-use map, urban functional area identification and natural resource management.

## 2. Materials and methods

**Fig 1:**
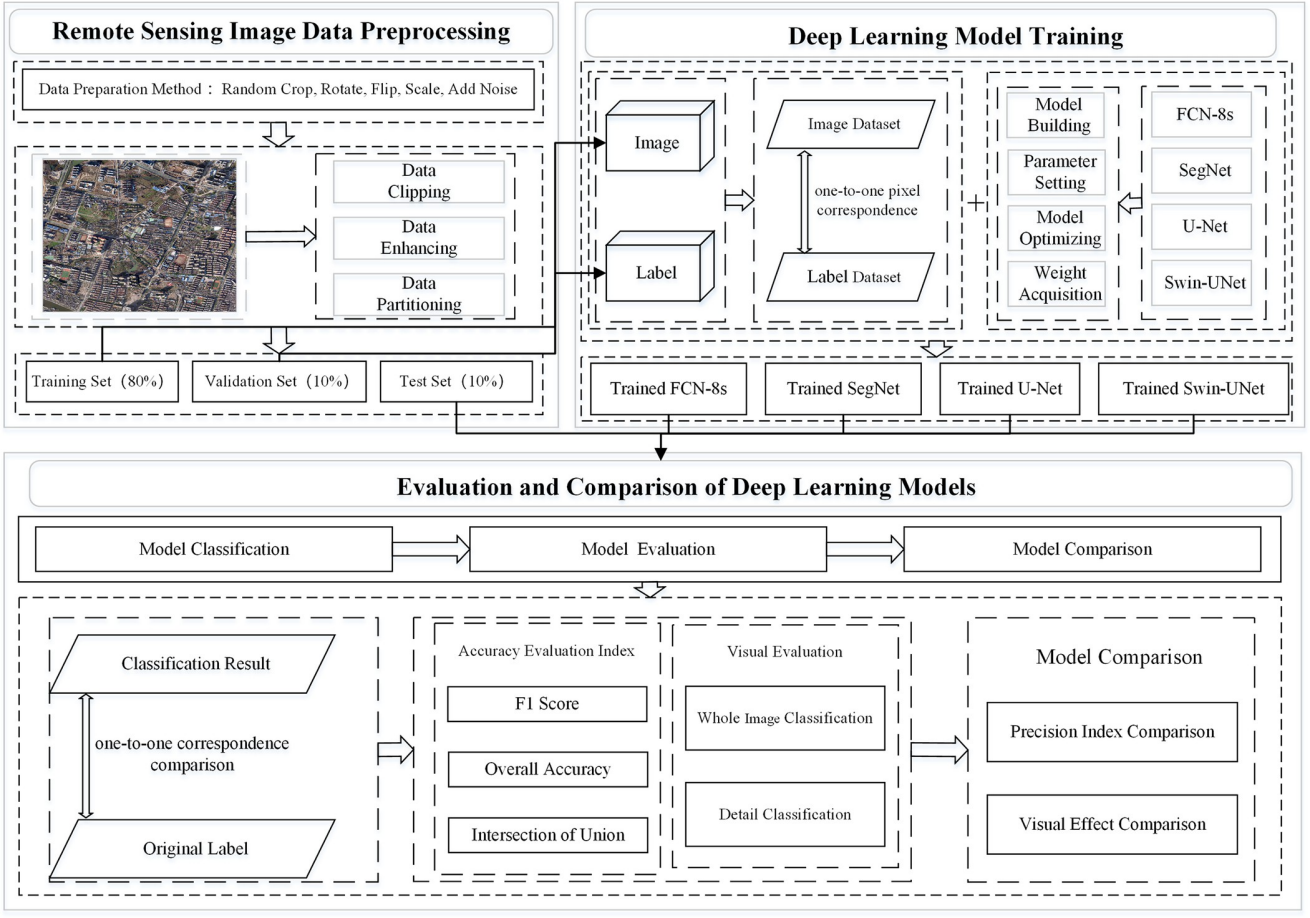
Technical flowchart of this study.

In this study, via experiments, four types of deep learning models, FCN-8s, SegNet, U-Net and Swin-UNet were constructed under an open-source, remote sensing image dataset to classify the land use types of remote sensing images. By analyzing the classification results of various models, we compare the differences in these four types of models. The technical flowchart of this study is shown in Fig 1.

### 2.1. Dataset

The data used in the experiment was obtained from the “AI classification and recognition of satellite images” competition in the 2017 Big Data and Computing Intelligence Contest (BDCI). The images in the dataset reflect an urban rural area in southern China in 2015, the specific location information of which is currently unavailable due to the organizer’s privacy protection. In this study, we choose this dataset for the following two reasons: (1) The data depicts remarkable details of the ground features with sub-meter accuracy. (2) This dataset removes redundant information irrelevant to classification, such as coordinate information, enabling the model to focus on nothing other than the ground objects during the training process. In this dataset, we obtain 5 large remote sensing images of unmanned aerial vehicles (UAVs), each of which is not equal in size; the overall size ranges from 4000×2000 to 8000×8000. The original remote sensing image is a 16-bit deep PNG image, and the labelled image is an 8-bit deep PNG image. In terms of spatial resolution, the spatial resolution of the image reached 1 meter. In terms of bands, each image has only three bands (R, G, and B). The training data in this dataset are annotated into five categories: vegetation, path, architecture, water body, and other.

### 2.2. Fully Convolutional Network (FCN-8s)

Since the fully convolutional neural network was proposed in 2015, image semantic segmentation has made great progress [36]. This network implements a fully convolutional network structure by replacing the fully connected layer in the CNN model with a 1×1 convolutional layer so that the single-vector output of the CNN can be densified to achieve dense classification [43]. Compared with traditional image segmentation methods, FCN-8s can produce quite good results through end-to-end training. Both learning and inference are performed in whole-image-at-a-time by dense feedforward computation and backpropagation. In network upsampling layers, it enables pixelwise prediction to match the original input resolution. The FCN model is mainly divided into three parts: the first part uses VGG16 as the encoder to extract the original image features, the second part uses DeconvNet to upsample the feature images in the deconvolution layer, and the last part fuses shallow semantic information with deep semantic information using a skip structure. In this study, we use transfer learning to port the already trained VGG16 model to our model network. VGG-16 has 5 convolutional blocks, with each containing 2 or 3 convolutional layers of kernel 3×3 with a padding of 1 followed by a rectified linear unit (ReLU) and batch normalization (BN). Each convolution block is followed by a max-pooling layer of size 2×2. Therefore, at the end of the encoder, the feature maps are each $\frac{W}{32}\times \frac{H}{32}$, where the original image has a resolution of W×H. The main role of DeconvNet is a decoder for upsampling and classification. DeconvNet mainly transforms the feature image passed through the encoder into the final label and restores it to the original spatial resolution. In this study, we use 5 deconvolution layers after acquiring the feature map, whose parameters are symmetric to those of the encoder. In the last deconvolutional layer of the model, we specify that its number of channels is equal to the number of categories in the dataset. To achieve a better classification effect, the skip structure is adopted in this study, and both the shallow and the deep semantic informations in the decoder are fused via three skip structures to form the FCN-8s model. We use a softmax layer to compute the multinomial logistic loss, averaged over the whole patch:

$$Loss=\frac{1}{N}\sum_{i=1}^{N}\sum_{j=1}^{k}y_{j}^{i}\log(\frac{\exp\left(z_{j}^{i}\right)}{\sum_{j=1}^{k}z_{j}^{i}})$$ (Eq 1)

where N is the number of pixels in the input image, k is the number of classes, and for a specified pixel i, $y_{j}^{i}$ denotes its ground truth label, which is a sparse one-hot vector, and $\left(z_{1}^{i},\cdots ,z_{j}^{i}\right)$ is the prediction vector. We minimize the average pixelwise classification loss.

### 2.3. SegNet

The SegNet model is a fully convolutional encoder-decoder structure that consists of an encoder for high-level feature extraction, a corresponding decoder for low-level information reconstruction, and a pixelwise classifier for prediction [44]. The encoder converts high-dimensional vectors to low-dimensional vectors and realizes low-dimensional extraction of high-dimensional features. The encoder consists of multiple layers. Each layer contains 2 convolution kernels of size 3×3, padding with the same size, followed by a ReLU, BN, and a max pooling layer of size 2×2. Each time the image passes through an encoder layer group, the size of the image is reduced to half of the original size, which enables effective data compression and feature extraction by traversing all layer groups in turn. Passing through the max pooling layer captures more translation-invariance features but blurs the spatial information. Therefore, in the process of pooling, the maximum pooling index information is simultaneously recorded so that the spatial information can be saved. The decoder network structure can be regarded as the inverse process of the encoder network structure. Each layer group mainly consists of an upsampling layer followed by two convolutional layers, a ReLU and BN. When upsampling the feature image, the maximum pooling index recorded in the decoder is used to upsample to obtain a sparse feature image and then putting the feature image into the convolution layer to obtain a dense feature image. After all the decoder layer groups, the spatial resolution of the image is restored to that of the original image. The feature image is the input to the softmax layer, as well as the probability of each category in each pixel is obtained, and the final label of each pixel is the category with the maximum probability. The loss function of this model is the same as that of the FCN-8s model.

### 2.4. U-Net

The network structure of the U-Net model is based on the expansion and modification of the fully convolutional neural network. The network structure is clear and elegant in the shape of a U, which consists of two parts, namely, the contraction path on the left, which is used to obtain contextual information, and the expansion path on the right, which is used to accurately locate the target. The contraction path of the U-Net model is a typical convolutional structure, and its structure and the parameters of the selected convolutional layer are consistent with the SegNet model [45]. The expansion path upsamples the shrunken feature map to restore the original spatial resolution. Different from the SegNet model, the U-Net model saves the feature map before each pooling operation, and the saved feature map is passed to the expansion path of the corresponding scale through skip connections, providing feature in-formation of different scales for the expansion path. By means of skip connections, the spatial information loss caused by the reduction in resolution attributed to the max pooling operation is recovered, and features of different resolutions are provided for the expansion path. The acquired feature images are put into the softmax layer for classification. The loss function is consistent with the FCN-8s and SegNet models.

### 2.5. Swin-UNet

Swin-UNet is a semantic segmentation network model founded on the transformer architecture, characterized by several key attributes. It employs a symmetric encoder-decoder structure based on the Swin-Transformer block, enabling seamless information flow [46]. The model includes a patch extension layer, which eliminates the need for convolution or interpolation operations while enhancing feature dimensionality and upsampling capabilities. Moreover, Swin-UNet merges skip connections with the transformer architecture, featuring components such as the Encoder, Bottleneck, Decoder, and skip connections. Its overall structure closely resembles the U-Net framework, with the notable difference being the integration of Swin-Transformer blocks.

### 2.6. Image preprocessing and experimental environment

Image preprocessing mainly includes three steps: (1) image cropping, (2) data augmentation, and (3) dataset division. The random cropping method was used to crop the images. We randomly generated 6000 (x, y) coordinate pairs for each image, and cropped an image of size 256×256 for each coordinate pair, and obtained a total of 30000 images. Afterward, we performed data augmentation operations on these 30,000 images. The data augmentation work mainly consists of 5 parts: (1) adding noise to the image, (2) blurring the image, (3) modifying the lighting conditions of the image, (4) mirroring the images and labels along the y-axis, (5) rotating the image and label, and setting the angle at 90°, 180° and 270°, respectively. We divided the generated dataset into a training set, a validation set and a test set according to the ratio of 8:1:1. The training set comprises 24,000 images which were random selected from the generated dataset for training the parameters in the model network. After allocating the training set, we randomly selected 3000 pictures from the remaining 6000 pictures for the validation set, in order to evaluate the training effect of the model to optimize the parameters. The remaining 3000 images were used as a test set to assess the performance of the model. The software conditions include Python 3.8, TensorFlow 2.5, and Cuda 11.2. The editor is Jupyter notebook. The hardware conditions are the Intel(R) Xeon CPU, (R) W-2245 CPU @ 3.90 GHz (16 CPUs), and NVIDIA Quadro RTX4000 GPU.

### 2.7. Evaluation indicators

This study uses the following evaluation metrics to evaluate the performance of the above three models on this dataset, namely, the F1 score, overall accuracy (OA), category pixel accuracy (CPA) and intersection of union (IOU).

The overall accuracy (OA) and category pixel accuracy (CPA) are very important evaluation indicators in the semantic segmentation model. The overall accuracy reflects the overall image classification effect of the model, and the category pixel accuracy reflects the classification effect of the model on various types of objects. The function expression is defined as follows:

$$OA=\frac{TP+TN}{TP+FN+FP+TN}$$ (Eq 2)

$$CPA=\frac{TP}{TP+FP}$$ (Eq 3)

The F1 score, a comprehensive evaluation metric, is defined as the harmonic mean of precision (P) and recall (R):

$$F1=\frac{2∙P∙R}{P+R}$$ (Eq 4)

$$P=\frac{TP}{TP+FP}$$ (Eq 5)

$$R=\frac{TP}{TP+FN}$$ (Eq 6)

The intersection and union (IOU) is an important evaluation indicator of semantic segmentation models that refers to the ratio of the intersection and union between the pixel set occupied by the real object image and the pixel set predicted by the model. The average intersection-over-union ratio (MIOU) is obtained by adding and averaging the IOUs predicted by various types of ground objects based on the model. The frequency-weighted intersection and union (FMIOU) sets weights for different objects according to the difference in the frequency of occurrence of various objects in an image, multiplies the weights with their corresponding IOU, and obtains the average value. The functional expressions of these three types of intersection ratios are presented as follows:

$$IoU=\frac{TP}{TP+FP+FN}$$ (Eq 7)

$$MIoU=\frac{\left[\frac{TP}{TP+FP+FN}+\frac{TN}{TN+FN+FP}\right]}{2}$$ (Eq 8)

$$FMIoU=\frac{TP+FN}{TP+FP+TN+FN}*\frac{TP}{TP+FP+FN}$$ (Eq 9)

There are four symbols in the above expressions, namely, TP, FP, FN, and TN. These four symbols indicate that the predicted positive samples are positive, that the predicted positive samples are negative, that the predicted negative samples are positive, and that the predicted negative samples are negative.

## 3. Results

### 3.1. The training process of deep learning models

To ensure a more objective comparison of experimental results, we conducted multiple iterations of the experiment and fine-tuned hyperparameters to optimize the experimental environment. The selected hyperparameters were as follows: (1) an initial learning rate of 0.001, (2) Adam as the model optimizer, (3) a batch size of 32 images, and (4) a callback function that adjusted the learning rate based on the training set’s accuracy. Specifically, if the training set’s accuracy did not improve for five consecutive epochs, the learning rate was halved, and training was terminated when the accuracy on the training set did not improve for 20 consecutive epochs.

**Fig 2:**
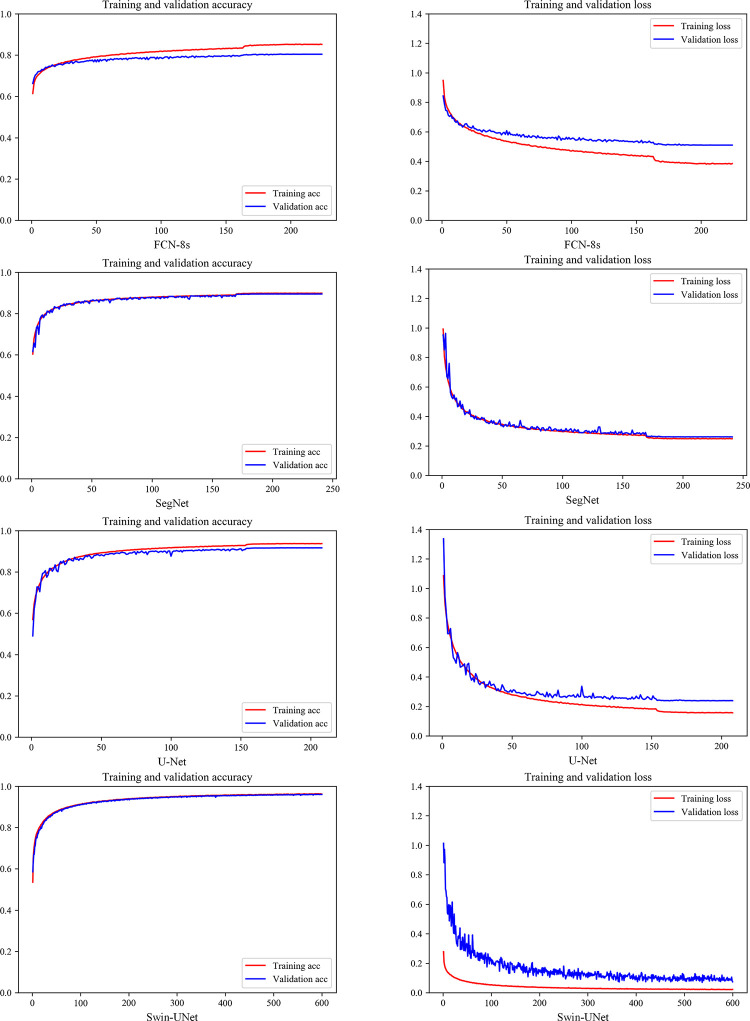
The training process of deep learning models.

Fig 2 illustrates the training progress of the FCN-8s, SegNet, U-Net, and Swin-UNet models. After a single epoch of training, the accuracy rates on the training set for the FCN-8s, SegNet, U-Net, and Swin-UNet models were 61.41%, 60.41%, 56.93%, and 53.54%, respectively. On the validation set, the accuracy rates were 66.26%, 61.65%, 49.03%, and 58.59%, respectively. When the models achieved their best training performance, the accuracy rates on the training set for the FCN-8s, SegNet, U-Net, and Swin-UNet models were 85.19%, 89.84%, 93.74%, and 96.25%, respectively, while the validation set accuracy rates were 80.41%, 89.49%, 91.68%, and 96.03%, respectively. Subsequently, we evaluated the classification performance of the trained models on the test set. The classification accuracies of FCN-8s, SegNet, U-Net, and Swin-UNet on the test set were 80.73%, 89.86%, 91.90%, and 96.01%, respectively. The Swin-UNet model exhibited the highest classification accuracy when the models were fully trained.

### 3.2. Quantitative evaluation

**Fig 3:**
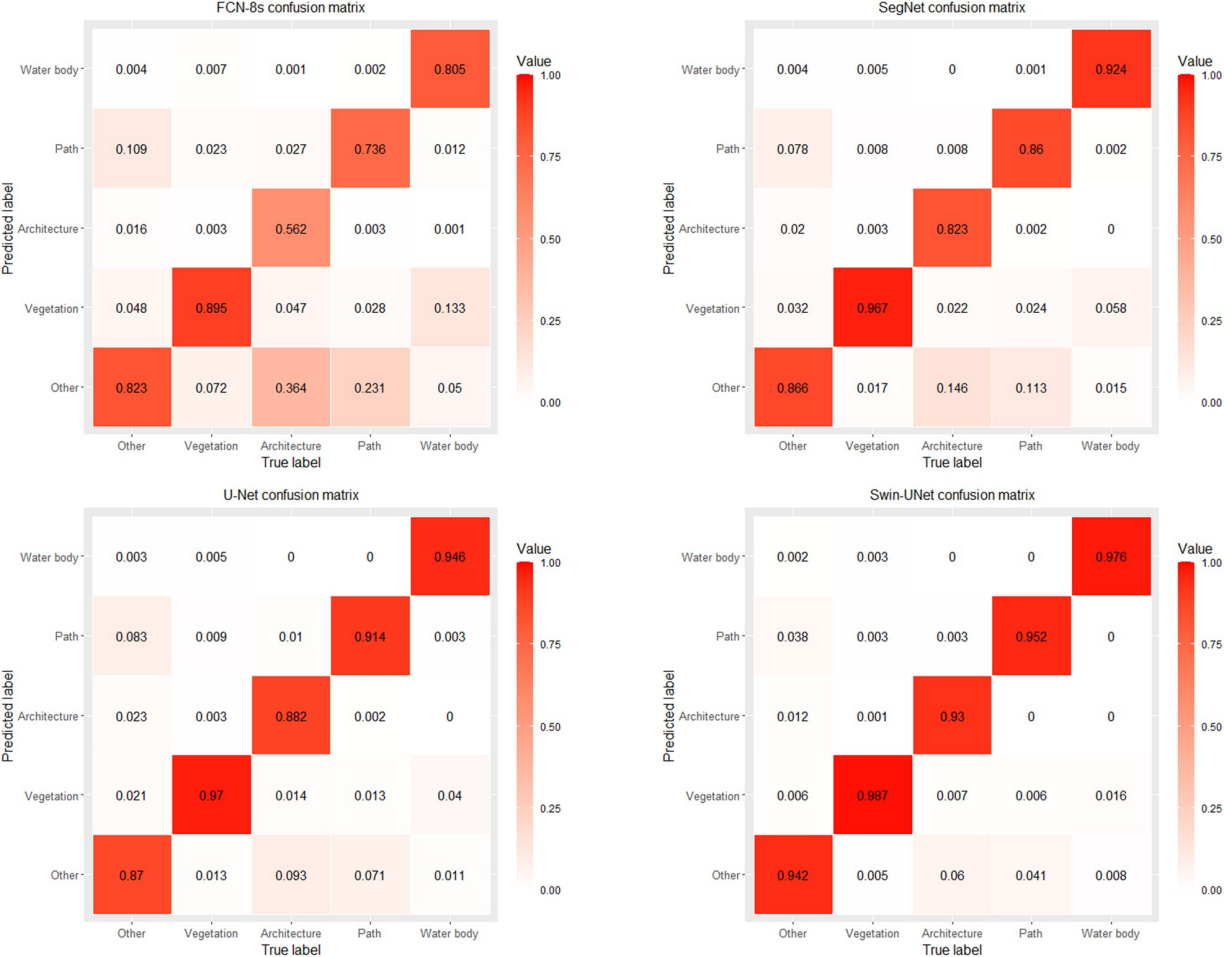
The confusion matrixes for the deep learning models.

Fig 3 presents the confusion matrix depicting the classification results of the four types of deep learning models on the test set. An observation of the confusion matrix reveals high values along the diagonal, indicating excellent classification performance by these models. Among these models, vegetation emerges as the most effectively classified feature. Progressing from FCN-8s to Swin-UNet, the classification accuracy for vegetation is 0.895, 0.967, 0.970, and 0.987, respectively. On the other hand, architectural features exhibit the lowest classification accuracy, with FCN-8s at 0.562 and a misclassification rate of 0.364 for other feature types. In contrast, SegNet, U-Net, and Swin-UNet demonstrate significant improvements in architectural classification accuracy at 0.823, 0.882, and 0.930, respectively, and a reduced misclassification rate for other features at 0.146, 0.093, and 0.060, respectively.

Table 1 quantitatively reflects the classification performance of these four models using metrics such as OA, CPA, F1 scores, IOU, MIOU, and FMIOU. When examining CPA for various land features, the ranking from highest to lowest is Swin-UNet, U-Net, SegNet, and FCN-8s. The most significant difference can be observed in the CPA for architectural, with a substantial 36.81% gap between Swin-UNet and FCN-8s, emphasizing Swin-UNet’s superior ability in recognizing complex features. Furthermore, considering the non-uniformity of ground objects in our study sample, we used the F1 score to correct for pixel accuracy. Comparative analysis reveals that, apart from FCN-8s, the F1 scores and CPA differences among various models are relatively small, indicating that Swin-UNet, U-Net, and SegNet can adapt well to biased samples, while FCN-8s is less suitable for such samples. Observing the IOU index, it’s evident that the IOU index decreases from Swin-UNet to FCN-8s, reflecting a reduction in model positioning accuracy and a weakening in classification performance. Overall, concerning OA indicators, Swin-UNet excels, surpassing FCN-8s, SegNet, and U-Net by 15.3%, 6.17%, and 4.13%, respectively. For the MIOU indicator, it surpasses them by 25.57%, 10.6%, and 7.16%, respectively. In terms of the FMIOU indicator, the lead is by 24.18%, 10.64%, and 7.25%, respectively. In summary, from a quantitative evaluation perspective, Swin-UNet demonstrates the highest classification performance, followed by U-Net, SegNet, and FCN-8s.

**Table 1:** Comparison of classification results of samples for test with different deep learning models.

Feature type	indicators	FCN-8s	SegNet	U-Net	Swin-UNet
Other	CPA	82.28%	86.62%	87.02%	94.20%
	F1 score	75.76%	86.22%	88.65%	94.40%
	IOU	60.98%	75.77%	79.61%	89.39%
Vegetation	CPA	89.51%	96.72%	96.99%	98.66%
	F1 score	89.59%	95.21%	96.48%	98.58%
	IOU	81.14	90.86%	93.20%	97.20%
Architecture	CPA	56.15%	82.33%	88.23%	92.96%
	F1 score	64.48%	84.95%	87.75%	93.26%
	IOU	50.92%	73.83%	78.18%	87.37%
Path	CPA	73.62%	86.00%	91.39%	95.22%
	F1 score	76.90%	87.24%	89.88%	95.01%
	IOU	62.46%	77.36%	81.62%	90.50%
Water body	CPA	80.46	92.41%	94.55%	97.55%
	F1 score	86.48%	94.01%	95.38%	97.48%
	IOU	76.19%	88.70%	91.16%	95.08%
Average	OA	80.73%	89.86%	91.90%	96.01%
	MIOU	66.34%	81.31%	84.75%	91.91%
	FMIOU	68.26%	81.80%	85.19%	92.44%

### 3.3. Visual classification evaluation

**Fig 4:**
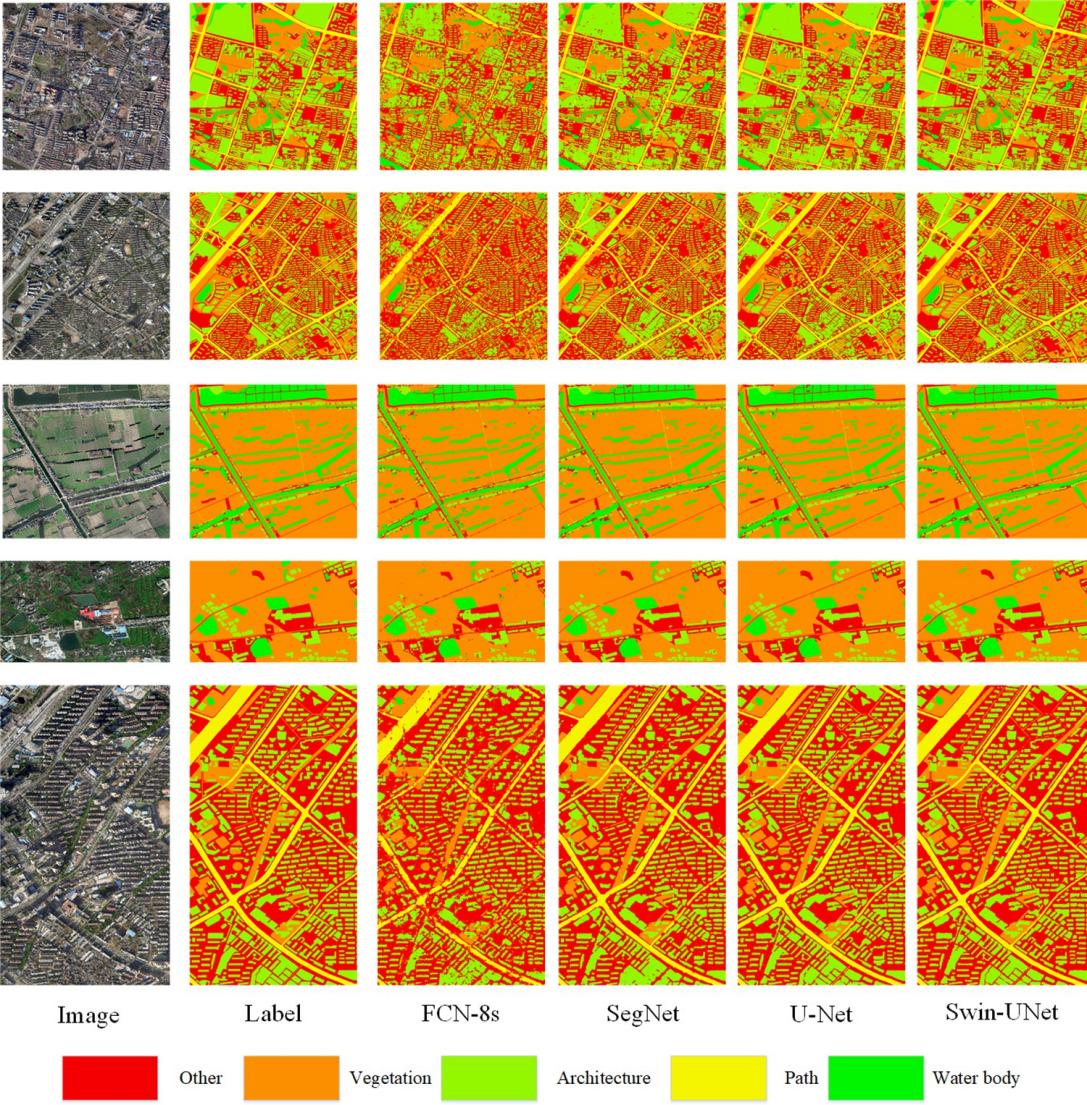
Classification diagram of various models.

**Fig 5:**
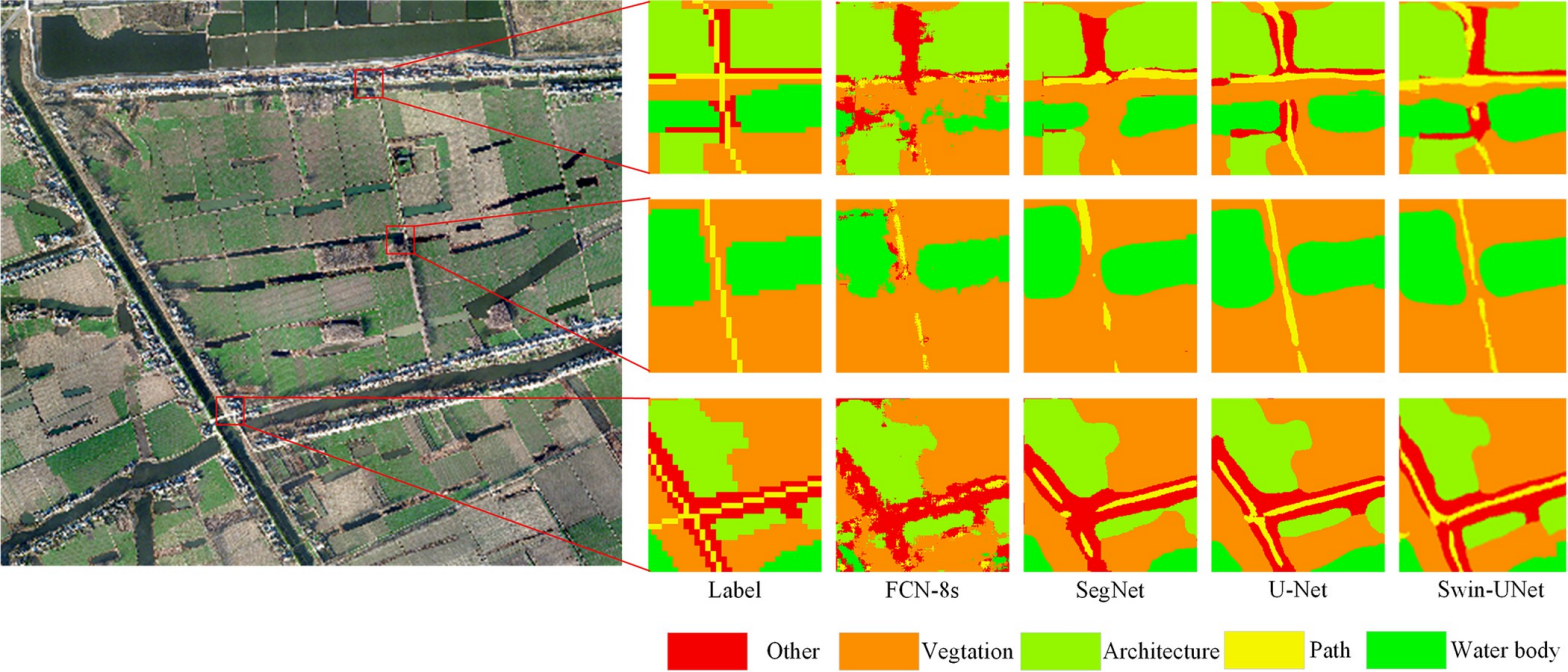
Comparison of the details of various models.

To gain a more intuitive understanding of the model’s classification performance, we applied the models to classify images from the original dataset, and the results are presented in Fig 4. In terms of the overall visual effect, all four deep learning models demonstrate the ability to effectively classify remote sensing images. However, significant differences emerge in the detailed predictions made by each model. The FCN-8s model, for example, produces intermittent predictions, particularly in its treatment of path features. While the SegNet model’s classification performance on roads is also suboptimal, it exhibits fewer issues compared to FCN-8s. U-Net excels in capturing finer details compared to SegNet. Notably, Swin-UNet’s predictions are exceptionally close to the actual labels, with minimal discrepancies. As illustrated in Fig 5, taking a microscopic view, FCN-8s and SegNet result in a spotted and intermittent classification of road features. U-Net outperforms both SegNet and FCN-8s, and Swin-UNet closely matches U-Net’s performance, producing predicted images that closely resemble the ground truth labels.

## 4. Discussion

In this study, we applied four types of semantic segmentation models with good applicability in land use classification to classify land use types for remote sensing images. In stark contrast to traditional physical models and conventional machine learning algorithms, deep learning networks rely exclusively on data for learning. Employing an end-to-end training approach, these models can adeptly assimilate ground object features. Furthermore, we conducted comparative analyses between our findings and relevant research. For instance, Zhang and his team [47] utilized FCN-8s, SegNet, and U-Net models for binary segmentation of infrared images in photovoltaic panel error detection, achieving classification accuracies of 95.64%, 95.84%, and 96.11%, respectively. However, our research emphasizes on the classification of high-resolution remote images with a multitude of diverse and unbalanced elements. Here, U-Net outperforms SegNet and significantly surpasses FCN-8s, owing to its amalgamation of the strengths of both models. U-Net inherits the symmetrical encoder-decoder structure from SegNet and incorporates the skip structure of FCN-8s, enabling it to capture extensive spatial and semantic information, resulting in superior classification. Our comparisons also reveal that the complexity of the classification scenario magnifies differences between model performances. Notably, the deep learning model Swin-UNet, built on transformer architecture, outperforms the CNN model significantly. Here, this is attributed to the transformer’s superior adaptability to extensive data, as its parameters dynamically evolve with output results, enhancing parameter extraction [48, 49]. Moreover, a single-layer transformer effectively fuses all information for decision-making, while a CNN relies on multiple layers and downsampling, sacrificing resolution and thereby falling short of the transformer’s classification prowess [50].

As the complexity of features in remote sensing images continues to grow, traditional physical models and machine learning approaches such as decision trees, support vector machines, and artificial neural networks are increasingly inadequate for object classification. These methods rely on simple thresholding to detect object edges, making them suitable only for distinguishing basic objects and often resulting in images with a fragmented, salt-and-pepper appearance [15]. This study underscores the deep learning network’s exceptional accuracy and comprehensive performance in classifying ground objects, achieving a comparatively low misclassification rate. When compared to conventional land use classification models, deep learning networks prove to be better suited for the classification of high-resolution remote sensing images with intricate features. In addition, we explored the distinctions between deep learning and shallow learning within this context. We designed a shallow learning model comprising just two convolutional and two deconvolutional layers, significantly reducing the model’s depth. Through experimentation, we discovered that the shallow learning model’s performance lagged even further behind FCN-8s, the least effective model among the three, with an accuracy gap of approximately 10 percentage points. The training graph for the shallow learning model is available in S1 Fig.

Remote sensing researchers are currently pursuing the self-adaptive interpretation of remote sensing images. As one of the mainstream methods for such exploration, the deep learning models require a large amount of data to adequately learn the characteristics of various ground objects [51]. Therefore, the efficiency of such models can be largely restrained by poor data quality when learning various features, whose classification effect can be even inferior to that of traditional physical models or machine learning models [52]. Given that the deep learning model is a black box model whose prediction effect cannot yet be fully determined, we might have problems explaining the model classification performance when using a complete deep learning model to classify land use types. Therefore, instead of solely relying on such models, we should use them as tools to assist our judgement to better classify land use types of remote sensing images. In terms of the latest development of remote sensing deep learning classification models, research has mostly focused on obtaining more detailed land cover information. For example, Liu et al. developed the multi-scale remote sensing classification model MS-GeoNet, which emphasizes on analyzing remote sensing images at multiple scales to obtain more land cover features [53]. However, geological knowledge remains the core of remote sensing interpretation [54]. Therefore, we improve the deep learning model by building a geoscience knowledge map so that by combining the data and knowledge, we can better complete the classification of remote sensing images. In summary, though having obvious advantages in high-resolution remote sensing image classification, the deep learning models are so far entirely data-driven that require further study on their data requirements and interpretability. Through this study, we believe after combining knowledge and data by building a geoscience knowledge graph and coupling a deep learning model, it can be a promising direction for future research.

## 5. Conclusion

In this study, we applied four well-established and robust deep learning models (FCN-8s, SegNet, U-Net, and Swin-UNet) to an open benchmark high-resolution remote sensing dataset. Our primary goal was to evaluate their image classification performance, utilizing metrics such as Intersection of Union, F1 score, and CPA. The key findings from our research are as follows: the overall accuracy on the test set for FCN-8s, SegNet, U-Net, and Swin-UNet reached 80.73%, 89.86%, 91.90%, and 96.01%, respectively. These models demonstrated their strongest classification performance in vegetation, while their performance was notably poorer for architectural elements. Moreover, when considering metrics like OA, CPA, IOU, F1 scores, MIOU, and FMIOU, it became evident that Swin-UNet displayed superior generalization abilities for complex terrains in comparison to the U-Net and SegNet models, significantly outperforming the FCN-8s model. The Swin-UNet model excelled in scenarios involving imbalanced data and more complex terrain, followed by the U-Net model and SegNet model, while the FCN-8s model was found to be less suitable for imbalanced data and demonstrated poor classification performance for complex terrain.

## Supporting information

S1 Fig The training process of shallow learning models. (TIF)

## References

[1] Coutts A.M.; Harris R.J.; Phan T.; Livesley S.J.; Williams N.S.G.; Tapper N.J. Thermal infrared remote sensing of urban heat: Hotspots, vegetation, and an assessment of techniques for use in urban planning. *Remote Sensing of Environment* 2016, 186, 637–651.

[2] Codemo A.; Pianegonda A.; Ciolli M.; Favargiotti S.; Albatici R. Mapping Pervious Surfaces and Canopy Cover Using High-Resolution Airborne Imagery and Digital Elevation Models to Support Urban Planning. *Sustainability* 2022, 14, 6149.

[3] Bastin G.; Scarth P.; Chewings V.; Sparrow A.; Denham R.; Schmidt M.; et al. Separating grazing and rainfall effects at regional scale using remote sensing imagery: A dynamic reference-cover method. *Remote Sensing of Environment* 2012, 121, 443–457.

[4] Chen B.; Tu Y.; Song Y.; Theobald D.M.; Zhang T.; Ren Z.; et al. Mapping essential urban land use categories with open big data: Results for five metropolitan areas in the United States of America. *ISPRS Journal of Photogrammetry and Remote Sensing* 2021, 178, 203–218.

[5] Bandyopadhyay K.; Sahoo R.; Singh R.; Pradhan S.; Singh S.; Krishna G.; et al. Characterization and crop planning of rabi fallows using remote sensing and GIS. *Current Science* 2015, 2051–2062.

[6] Zudilin S.; Iralieva Y.S. Automation of land use planning based on geoinformation modeling. *In Proceedings of the IOP Conference Series*: *Earth and Environmental Science* 2021, 720, 012039.

[7] Yuan F. Land-Cover Change And Environmental Impact Analysis In The Greater Mankato Area Of Minnesota Using Remote Sensing And Gis Modelling. *Int J Remote Sens* 2008, 29, 1169–1184.

[8] Zhang C.; Pan X.; Li H.; Gardiner A.; Sargent I.; Hare J.; et al. A hybrid MLP-CNN classifier for very fine resolution remotely sensed image classification. *Isprs Journal of Photogrammetry & Remote Sensing* 2018, 140, 133–144.

[9] Feng Q.; Yang J.; Zhu D.; Liu J.; Guo H.; Bayartungalag B.; et al. Integrating multitemporal Sentinel-1/2 data for coastal land cover classification using a multibranch convolutional neural network: A case of the Yellow River Delta. *Remote Sensing* 2019, 11, 1006.

[10] Zhu Q.; Zhong Y.; Liu Y.; Zhang L.; Li D. A Deep-Local-Global Feature Fusion Framework for High Spatial Resolution Imagery Scene Classification. *Remote Sensing* 2018, 10, 568.

[11] Huang C.; Davis L.S.; Townshend J.R.G. An assessment of support vector machines for land cover classification. *Int J Remote Sens* 2002, 23, 725–749.

[12] Pal M.; Mather P.M. Support vector machines for classification in remote sensing. *Int J Remote Sens* 2005, 26, 1007–1011.

[13] Pal M. Random forest classifier for remote sensing classification. *Int J Remote Sens* 2005, 26, 217–222.

[14] Otukei J.R.; Blaschke T.; Woldai T.; Annegarn H. Land cover change assessment using decision trees, support vector machines and maximum likelihood classification algorithms. *International Journal of Applied Earth Observations & Geoinformation* 2010, 12, S27–S31.

[15] Duro D.C.; Franklin S.E.; Dubé M.G. A comparison of pixel-based and object-based image analysis with selected machine learning algorithms for the classification of agricultural landscapes using SPOT-5 HRG imagery. *Remote Sensing of Environment* 2012, 118, 259–272.

[16] Rahman M.R.; Saha S.K. Multi-resolution segmentation for object-based classification and accuracy assessment of land use/land cover classification using remotely sensed data. *Journal of the Indian Society of Remote Sensing* 2008, 36, 189–201.

[17] Lka D.; Maier B.; Seijmonsbergen A.C. Improved Landsat-based forest mapping in steep mountainous terrain using object-based classification. *Forest Ecology &* *Management* 2003, 183, 31–46.

[18] Peña-Barragán J.M.; Ngugi M.K.; Plant R.E.; Six J. Object-based crop identification using multiple vegetation indices, textural features and crop phenology. *Remote Sensing of Environment* 2011, 115, 1301–1316.

[19] Yan G.; Mas J.F.; Maathuis B.H.P.; Xiangmin Z.; Dijk P.M.V. Comparison of pixel‐based and object‐oriented image classification approaches—a case study in a coal fire area, Wuda, Inner Mongolia, China. *Int J Remote Sens* 2006, 27, 4039–4055.

[20] Myint S.W.; Gober P.; Brazel A.; Grossman-Clarke S.; Weng Q. Per-pixel vs. object-based classification of urban land cover extraction using high spatial resolution imagery. *Remote Sensing of Environment* 2013, 115, 1145–1161.

[21] Costa H.; Foody G.M.; Boyd D.S. Supervised methods of image segmentation accuracy assessment in land cover mapping. *Remote Sensing of Environment* 2018, 205, 338–351.

[22] Wei W.; Zhang J.; Zhang L.; Tian C.; Zhang Y. Deep Cube-Pair Network for Hyperspectral Imagery Classification. *Remote Sensing* 2018, 10, 783.

[23] Li Y.; Qi H.; Dai J.; Ji X.; Wei Y. Fully Convolutional Instance-Aware Semantic Segmentation. *Proceedings of the IEEE conference on computer vision and pattern recognition* 2016, *pp*, 2359–2369.

[24] He Z.; Liu H.; Wang Y.; Hu J. Generative Adversarial Networks-Based Semi-Supervised Learning for Hyperspectral Image Classification. *Remote Sensing* 2017, 9, 1042.

[25] Yang J.; Zhao Y.Q.; Chan J. Hyperspectral and Multispectral Image Fusion via Deep Two-Branches Convolutional Neural Network. *Remote Sensing* 2018, 10, 800.

[26] Mei S.; Yuan X.; Ji J.; Zhang Y.; Wan S.; Du Q. Hyperspectral Image Spatial Super-Resolution via 3D Full Convolutional Neural Network. *Remote Sensing* 2017, 9, 1139.

[27] Yue J.; Zhao W.; Mao S.; Liu H. Spectral–spatial classification of hyperspectral images using deep convolutional neural networks. *Remote Sensing Letters* 2015, 6, 468–477.

[28] Couliably S.; Kamsu-Foguem B.; Kamissoko D.; Traore D. Explainable deep convolutional neural networks for insect pest recognition. *Journal of Cleaner Production* 2022, 371, 133638.

[29] Li H.; Fu K.; Yan M.; Sun X.; Sun H.; Diao W. Vehicle detection in remote sensing images using denoizing-based convolutional neural networks. *Remote Sensing Letters* 2017, 8, 262–270.

[30] Gallego A.J.; Pertusa A.; Gil P. Automatic Ship Classification from Optical Aerial Images with Convolutional Neural Networks. *Remote Sensing* 2018, 10, 511.

[31] Chen S.; Zhan R.; Zhang J. Geospatial Object Detection in Remote Sensing Imagery Based on Multiscale Single-Shot Detector with Activated Semantics. *Remote Sensing* 2018, 10, 820.

[32] Zheng Z.; Zhong Y.; Su Y.; Ma A. Domain Adaptation via a Task-Specific Classifier Framework for Remote Sensing Cross-Scene Classification. *IEEE Transactions on Geoscience and Remote Sensing* 2022, 60, 1–13.

[33] Hu F.; Xia G.S.; Hu J.; Zhang L. Transferring Deep Convolutional Neural Networks for the Scene Classification of High-Resolution Remote Sensing Imagery. *Remote Sensing* 2015, 7, 14680–14707.

[34] Zhong Y.; Fei F.; Liu Y.; Zhao B.; Jiao H.; Zhang L. SatCNN: satellite image dataset classification using agile convolutional neural networks. *Remote Sensing Letters* 2017, 8, 136–145.

[35] Zhang S.; Li C.; Qiu S.; Gao C.; Zhang F.; Du Z.; et al. EMMCNN: An ETPS-based multi-scale and multi-feature method using CNN for high spatial resolution image land-cover classification. *Remote Sensing* 2019, 12, 66.

[36] Long J.; Shelhamer E.; Darrell T. Fully convolutional networks for semantic segmentation. *In Proceedings of the Computer Vision and Pattern Recognition* 2015, *pp*, 3431–3440. 10.1109/TPAMI.2016.2572683 27244717

[37] Fu G.; Liu C.; Zhou R.; Sun T.; Zhang Q. Classification for High Resolution Remote Sensing Imagery Using a Fully Convolutional Network. *Remote Sensing* 2017, 9, 498.

[38] Marmanis D.; Schindler K.; Wegner J.D.; Galliani S.; Datcu M.; Stilla U. Classification With an Edge: Improving Semantic Image Segmentation with Boundary Detection. *Isprs Journal of Photogrammetry &* *Remote Sensing* 2016, 135, 158–172.

[39] Audebert N.; Saux B.L.; Lefèvre S. Beyond RGB: Very High Resolution Urban Remote Sensing With Multimodal Deep Networks. *Isprs Journal of Photogrammetry &* *Remote Sensing* 2017, 140, 20–32.

[40] Persello C.; Tolpekin V.; Bergado J.R.; De By R. Delineation of agricultural fields in smallholder farms from satellite images using fully convolutional networks and combinatorial grouping. *Remote sensing of environment* 2019, 231, 111253. doi: 10.1016/j.rse.2019.111253 31534278 PMC6737917

[41] Jeppesen J.H.; Jacobsen R.H.; Inceoglu F.; Toftegaard T.S. A cloud detection algorithm for satellite imagery based on deep learning. *Remote sensing of environment* 2019, 229, 247–259.

[42] Yao Junyuan, and Jin Shuanggen. Multi-category segmentation of Sentinel-2 images based on the Swin UNet method[J]. *Remote Sensing* 2022, 14 *(*14*)*: 3382.

[43] Audebert N, Le Saux B, Lefèvre S. Beyond RGB: Very high resolution urban remote sensing with multimodal deep networks. *ISPRS journal of photogrammetry and remote sensing* 2018, 140, 20–32.

[44] Badrinarayanan V.; Kendall A.; Cipolla R. Segnet: A deep convolutional encoder-decoder architecture for image segmentation. *IEEE transactions on pattern analysis and machine intelligence* 2017, 39, 2481–2495. doi: 10.1109/TPAMI.2016.2644615 28060704

[45] Ronneberger, O.; Fischer, P.; Brox, T. U-Net: Convolutional Networks for Biomedical Image Segmentation. *Medical Image Computing and Computer-Assisted Intervention–MICCAI 2015*: *18th International Conference* 2015, 8, 234–241.

[46] Cao H.; Wang Y.; Chen J.; Jiang D.; Zhang X.; Tian Q.; et al. Swin-unet: Unet-like pure transformer for medical image segmentation. *European conference on computer vision* 2022, *pp*, 205–218.

[47] Zhang, H.; Hong, X.; Zhou, S.; Wang, Q. Infrared image segmentation for photovoltaic panels based on res-unet. *In Proceedings of the Chinese conference on pattern recognition and computer vision (PRCV)*, 2019, pp, 611–622.

[48] Hassani A.; Walton S.; Shah N.; Abuduweili A.; Li J.; Shi H. Escaping the big data paradigm with compact transformers. *arXiv preprint arXiv*:*210405704*. 2021.

[49] Zhang W.; Jiao L.; Liu F.; Yang S.; Liu J. Adaptive contourlet fusion clustering for SAR image change detection. *IEEE Transactions on Image Processing*. 2022, 31, 2295–308. doi: 10.1109/TIP.2022.3154922 35245194

[50] Zhang J.; Liu Y.; Wu Q, Wang Y.; Liu Y.; Xu X.; et al. SWTRU: star-shaped window transformer reinforced U-net for medical image segmentation. *Computers in Biology and Medicine*. 2022, 150, 105954. doi: 10.1016/j.compbiomed.2022.105954 36122443

[51] Li X.; He M.; Li H.; Shen H. A combined loss-based multiscale fully convolutional network for high-resolution remote sensing image change detection. *IEEE Geoscience and Remote Sensing Letters* 2021, 19, 1–5.

[52] Wang Z.; Chen J.; Hoi S.C. Deep learning for image super-resolution: A survey. *IEEE transactions on pattern analysis and machine intelligence* 2020, 43, 3365–3387. 10.1109/TPAMI.2020.2982166 32217470

[53] Liu T, Yao L, Qin J, Lu N, Jiang H, Zhang F, et al. Multi-scale attention integrated hierarchical networks for high-resolution building footprint extraction. *International Journal of Applied Earth Observation and Geoinformation* 2022, 109, 102768.

[54] Hao X.; Ji Z.; Li X.; Yin L.; Liu L.; Sun M.; et al. Construction and application of a knowledge graph. *Remote Sensing* 2021, 13, 2511.

