# Supplementary figures and images for: Land-use classification based on high-resolution remote sensing imagery and deep learning models
Source: PLoS One. 2024 Apr 18;19(4):e0300473. doi: 10.1371/journal.pone.0300473 (PMC11025814; doi:10.1371/journal.pone.0300473)

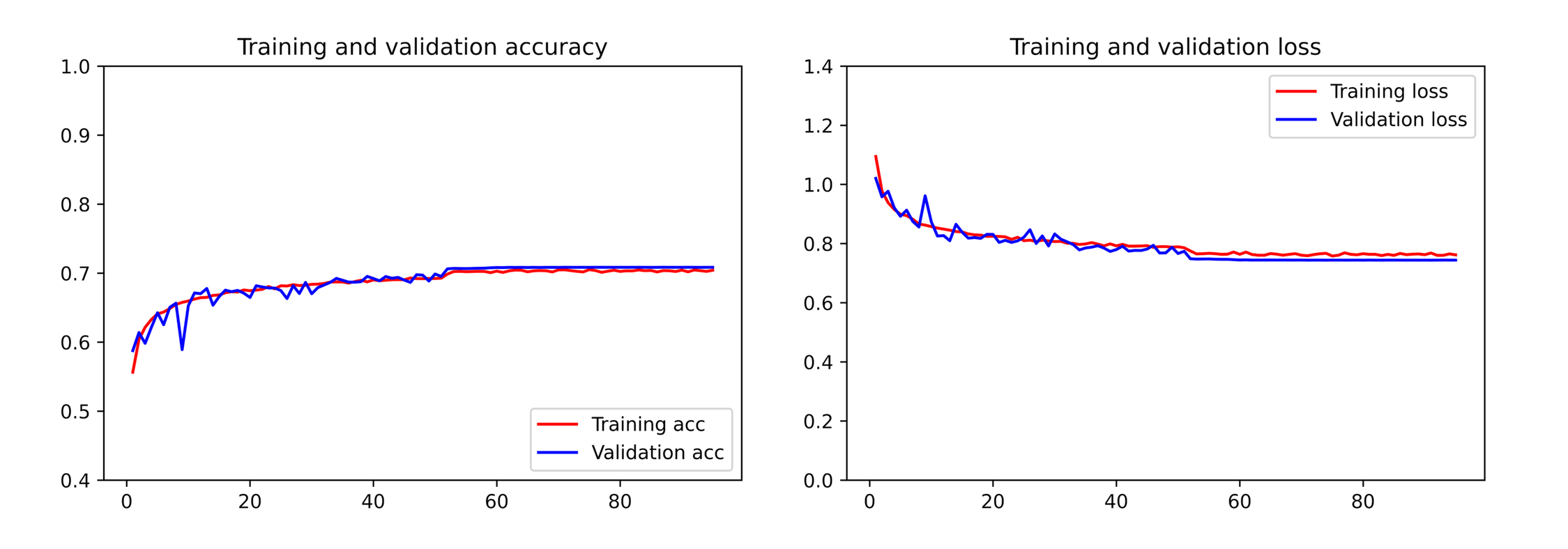

Supplement: S1 Fig — (TIF) [file pone.0300473.s001.tif]
